# From Child to Young Adult, the Brain Changes Its Connections

**DOI:** 10.1371/journal.pbio.1000158

**Published:** 2009-07-21

**Authors:** Richard Robinson

**Affiliations:** Freelance Science Writer, Sherborn, Massachusetts, United States of America

**Figure pbio-1000158-g001:**
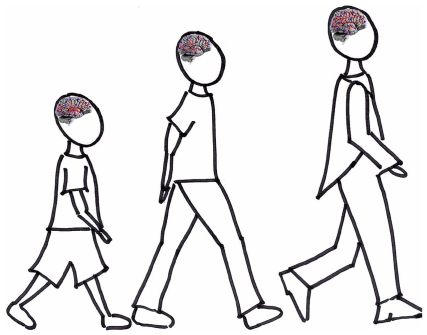
Brain rewires during development. (Image: Meghan Meyer).

[Fig pbio-1000158-g001]Complex enterprises of any sort—be they businesses or brains—involve the flow of lots of information. A central goal in understanding such enterprises is figuring out “who talks to whom.” A business may have to change its organizational flow chart as it matures in order to accommodate new challenges or weed out unprofitable sectors. Is the same true for brains? Do the networks of communication within the brain change as the brain matures?

That's the question Kaustubh Supekar, Mark Musen, and Vinod Menon set out to answer in this issue of *PLoS Biology*, by comparing patterns of activity in the brains of children ages 7 to 9 to those in young adults, ages 19 to 22. Many studies have shown massive structural changes in the brain between these ages—redundant synapses get pruned and long-distance axonal tracts get more heavily insulated with myelin. But the authors wanted to know whether these structural changes were accompanied by functional changes, so they turned to functional magnetic resonance imaging (fMRI), in which greater blood flow (a proxy for greater neuronal activity) makes for a brighter image. Regions that lit up at the same time, they reasoned, were involved in some type of coordinated activity. They imaged their subjects at rest, without giving them anything special to do or think about while lying in the machine, because they wanted to avoid any residual task-specific talk among different brain centers. The activity patterns the researchers observed, therefore, represented the basic functional connectivity, the underlying ongoing conversation, in the brains of their child or young-adult subjects. They also examined how functional connectivity changed with the physical distance along long-distance axonal tracts that wire the brain. To do so, they used diffusion tensor imaging (DTI), which provides quantitative information about fibers linking different parts of the brain.

As has been observed in older adults, both children and young adults manifested a “small world” type of functional architecture throughout the brain, with lots of densely connected local clusters of activity and short distances between most pairs of interacting structures. This type of connectivity is thought to increase local information processing efficiency. But beyond the structure of the local cluster, the two age groups differed significantly. The brains of young adults were more hierarchical and had more regions involved in larger and longer-distance clusters of activity. In a variety of information processing systems, hierarchy is thought to increase the ability of some structures to control others, but it comes with heightened vulnerability to a breakdown in communication. The lesser degree of hierarchy in children's brains may protect them from this vulnerability in these middle stages of the brain's development.

There were other differences as well. In children's brains, there were stronger and more abundant connections between subcortical and cortical regions, while in young-adult brains, the connections among cortical regions were more prominent. These changes parallel the age-dependent increase in myelination seen in the fiber bundles connecting these areas, confirming that the strengthening of these sets of connections has functional consequences and that, together, these comprise major events in the development of the brain.

Though more research will be needed to make the case, developmental changes in functional connectivity suggest several behavioral consequences. The critical subcortical regions linked up in the children's connection pattern include the basal ganglia, where reward is processed, leading to incentive-based learning and habit formation—fundamentally important processes in the young. Connections among the cortical areas seen in the young-adult pattern are likely to facilitate more flexible behaviors through integration of multiple higher brain inputs and processing of more cognitively demanding tasks. Finally, another area for further research will be to determine how these connectivity patterns are affected in behavioral and learning disorders, such as autism and attention deficit hyperactivity disorder, which accumulating evidence suggests include disruptions of normal communication patterns within the brain.

**Supekar K, Musen M, Menon V (2009) Development of Large-Scale Functional Brain Networks in Children. doi:10.1371/journal.pbio.1000157**

